# Digital Twins in Health Care: Ethical Implications of an Emerging Engineering Paradigm

**DOI:** 10.3389/fgene.2018.00031

**Published:** 2018-02-13

**Authors:** Koen Bruynseels, Filippo Santoni de Sio, Jeroen van den Hoven

**Affiliations:** Department of Philosophy, Faculty of Technology, Policy and Management, Delft University of Technology, Delft, Netherlands

**Keywords:** therapy, ethics of human enhancement, digital twins, privacy in healthcare technologies, value sensitive design in healthcare technologies, ethics of biomedical data, personalized medicine, virtual self

## Abstract

Personalized medicine uses fine grained information on individual persons, to pinpoint deviations from the normal. ‘Digital Twins’ in engineering provide a conceptual framework to analyze these emerging data-driven health care practices, as well as their conceptual and ethical implications for therapy, preventative care and human enhancement. Digital Twins stand for a specific engineering paradigm, where individual physical artifacts are paired with digital models that dynamically reflects the status of those artifacts. When applied to persons, Digital Twins are an emerging technology that builds on *in silico* representations of an individual that dynamically reflect molecular status, physiological status and life style over time. We use Digital Twins as the hypothesis that one would be in the possession of very detailed bio-physical and lifestyle information of a person over time. This perspective redefines the concept of ‘normality’ or ‘health,’ as a set of patterns that are regular *for a particular individual*, against the backdrop of patterns observed in the population. This perspective also will impact what is considered therapy and what is enhancement, as can be illustrated with the cases of the ‘asymptomatic ill’ and life extension via anti-aging medicine. These changes are the consequence of how meaning is derived, in case measurement data is available. Moral distinctions namely may be based on patterns found in these data and the meanings that are grafted on these patterns. Ethical and societal implications of Digital Twins are explored. Digital Twins imply a data-driven approach to health care. This approach has the potential to deliver significant societal benefits, and can function as a social equalizer, by allowing for effective equalizing enhancement interventions. It can as well though be a driver for inequality, given the fact that a Digital Twin might not be an accessible technology for everyone, and given the fact that patterns identified across a population of Digital Twins can lead to segmentation and discrimination. This duality calls for governance as this emerging technology matures, including measures that ensure transparency of data usage and derived benefits, and data privacy.

## Personalized Medicine – Therapy as Digitally Supported Engineering

Personalized medicine starts from the assumption that refined mathematical models of patients, fuelled by big biodata, will drive more precise and effective medical interventions. Instead of basing medical interventions on the responses of the average person, digital models now even carry the promise to tailor healthcare to the anticipated responses of *individual* patients. The availability of molecular readout technologies and of sufficient computational power increasingly makes it possible to build such personalized models, and to complement them with continuously tracked health and lifestyle parameters. This eventually can result in a digital representation of an individual patient – a ‘virtual patient’ or even an ‘in-silico-self.’ Such strategy was proposed as a venue for European healthcare: “realistic computer models that are built and validated upon experimental big data collected by the most advanced technologies from molecular to macroscopic scales” (Lehrach et al., 2016). This manifesto projects vast health improvements, reduction of health care costs, and an increased personal freedom in dealing with our own biology.

Provided such ‘virtual patients’ indeed become available, they will take the current engineering practices in health care to a different level. In this paper, we elaborate on the striking similarities between these emerging trends in health care, and the emerging concept of Digital Twins in engineering. A Digital Twin in engineering consists of a particular artifact and a computer model that closely reflects the state of that artifact. The artifact – for instance the engine of an airplane – and its model are closely coupled via a multitude of sensors. Such dynamic computer models prove to be very instrumental when doing predictive maintenance or engineering of real-world artifacts. At the instrumental level, a ‘virtual self’ of a patient conceptually is on a same par with a Digital Twin of a complex and mission critical artifact. Digital Twins therefore provide a conceptual instrument to analyze the impact of these novel engineering practices on core concepts in current debates on health care, like health, disease, preventative care, and enhancement. One can analyze these health care concepts in analogy with engineering concepts of ‘normal functioning,’ ‘malfunctioning,’ ‘predictive maintenance,’ ‘performance optimization,’ and the ‘implementation of new functionality.’

Engineering approaches in general are ubiquitous in modern medicine. In current health care practices, one engineers a vascular bypass to restore the blood flow in case of atherosclerosis, repairs a heart valve, or replaces and old lens in the eye of a patient suffering from cataract. These engineering practices are rooted in the explanatory power and practical successes of the mechanical philosophy that has gradually emerged since the Renaissance. For instance, the drainage of the Low Countries provided significant improvements in the understanding of pumps, valves and hydraulic systems. These evolutions resonated in the work of contemporaries that studied vascular anatomy and the working of the heart (Novell, 1990). The description of the heart as a pump with one-way valves eventually opened the route to engineering actions like heart valve replacement. The engineering perspective developed into an important paradigm in current health care and therapy. Many Technical Universities in the world now train and educate engineers in clinical technology curricula, and doctors routinely work with engineers with a range of different backgrounds.

This engineer’s point of view also forms the hidden premise in many debates about human enhancement. When it is possible to replace broken parts in the body, and to tweak, fine tune, and optimize them, it is in principle also possible to extend this body with new functionalities. Neural implants can for instance be used for visual prosthetics for blind people, but they also open the route toward capabilities going beyond normal human sight and give access to a range of normally inaccessible parts of the electromagnetic spectrum. Drugs like Ritalin can be used to help ADHD patients to focus, but can also be applied to boost mental performance in people that don’t suffer from ADHD. The engineer’s perspective becomes especially striking in the case of human germline editing with the aid of CRISPR/cas (Liang et al., 2015). In therapeutic applications, one could consider the editing of the nucleotides that give rise to severe Mendelian diseases, thereby preventing a lot of human suffering. With the same engineering approach, one can potentially bring in traits that go beyond current human capabilities. For example, one could consider engineering human hemoglobin to be more like shark-hemoglobin, thereby allowing humans to store more oxygen in the blood. Substantial engineering of traits will though be very difficult if not unfeasible. The engineering approach to health in contemporary medicine is confronted with the sheer complexity of the human body and its operations. Here a purely mechanistic approach proved to be insufficient. It is for instance very difficult or impossible to precisely predict the efficacy of a drug and its side effects in a concrete patient. A large quantity of the massively prescribed blockbuster drugs therefore has suboptimal effects. Complex multifactorial diseases prove to be very hard to tackle via an engineering approach. Along these lines, human enhancement will require the engineering of complex and interconnected traits. This might well be impossible to achieve with current medical engineering approaches.

To get a better grip on this complexity, large initiatives are established to generate detailed molecular data of patients and healthy research subjects. Publicly funded initiatives like Genomics England (The 100.000 Genomes Project, 2017) or the US precision medicine (PMI Working Group, 2015), and private initiatives like Human Longevity Inc. and the Mayo Clinic Centre for Individualized Medicine gather genomic information on large numbers of individuals. These initiatives ultimately aim at the development of digital models of certain aspects of patients, allowing for more targeted health care interventions. Instead of using an overall scheme of the average human body and its responses, personalized medicine starts from the premise that health care can vastly benefit from detailed molecular and life style data of each individual patient. In the case of picking the right drug to treat a cancer, the efficacy of this approach already has been proven. Genotyping an individual’s tumor tissue provides clues on which drug will result in the biggest impact and the smallest side effects (Kummar et al., 2015). Personalized medicine also carries the promise to lead to predictive medicine, where diseases can be predicted and thereby also preventatively treated. All these initiatives constitute steps in the direction of ‘virtual patients’: data-driven mathematical models of patients that allow for more precise and effective medical interventions. The modalities of how and where such patient models will reside, who will own these models and who will be able to access them, these all need to be determined as this emerging technology evolves. The choices made will have strong impacts on health care related values like data privacy and patient autonomy. Among the current implementations for instance are private/academic partnership where the company, and not the research subject owns the data, and in which research subjects are allowed a certain level of access to their data (Project Baseline, 2017).

The analogy with Digital Twins, as elaborated in this paper, provides a conceptual tool to pinpoint where the engineering paradigm holds true, and where it differs in case of personalized models of individuals. The detailed information contained in virtual selves will allow for a quantitative underpinning of medical engineering actions. But in contrast to the relation between an artifact and its digital representation, a person’s ‘virtual self’ does not only relate to the physicalist realm, but also to the realm of language and meaning. The handling of an artifact’s Digital Twin and a person’s ‘virtual self’ diverges at the point where meanings get attributed to features identified in the virtual representations. This will make that besides a quantitative aspect, also conceptual and ethical aspects come into play. We will analyze what implications a Digital Twin engineering paradigm in health care can entail.

## Digital Twins in Engineering Practices, and Their Relevance for Data- and Model-Driven Healthcare

Digital Twins-based practices in civil engineering provide a good conceptual framework, when analysing the impact of a data- and model-driven healthcare on concepts of health, disease, and enhancement.

Unlike traditional engineering models, Digital Twins reflect the particular and individual, the idiosyncratic. Traditional engineering models reflect the generic: they apply to multiple instances. A Computer Aided Design model of an airplane jet engine reflects the structure of all the jet engine instances that are built based on this model. A Digital Twin though tightly connects the physical system (e.g., one particular machine) with its computer model, so that the latter closely reflects the architecture, the dynamics *and* the actual state of this one particular system. Sensors that allow for continuous monitoring of technical systems increasingly make it possible to create such individualized dynamic models. This type of model has been termed ‘Digital Twin,’ since it closely represents the inner state of the physical twin object. Digital Twin models are used in predictive maintenance, where they are used to identify anomalies long before parts actually break down. Digital Twins are also used to simulate the outcome of technical interventions like fixes and upgrades. The Digital Twin concept for instance was applied by NASA in the development of aerospace vehicles that last longer and endure more extreme conditions. In this context, they were defined as “an integrated multi-physics, multi-scale, probabilistic simulation of an as-built vehicle or system that uses the best available physical models, sensor updates, fleet history, etc., to mirror the life of its corresponding flying twin. … By combining all of this information, the Digital Twin continuously forecasts the health of the vehicle or system, the remaining useful life and the probability of mission success. The Digital Twin can also predict system response to safety-critical events and uncover previously unknown issues before they become critical by comparing predicted and actual responses” (Glaessgen and Stargel, 2012). The concept emerges also as a key element in Industry 4.0 strategies. It was termed “a living model of the physical asset or system” that allows to “continually adapt to changes in the environment or operations and deliver the best business outcome” (Infosys Insights, 2016), a “digital copy that is created and developed simultaneously with the real machine” (Siemens, 2015), “the bridge from the physical to the digital worlds, providing understanding of each unique asset over time” (General Electric, 2017). Digital Twins have been applied to optimize the operations of power plants, wind turbine parks, critical jet engine components, etc.

The emerging data-driven personalized health care practices bear striking resemblances to Digital Twins driven engineering in industry. These novel engineering approaches to health care also build on dynamic and high resolution digital models of genetic, biochemical, physiological and behavioral aspects of individual persons. Digital Twin based medicine is far from being an established fact yet. Various initiatives nevertheless pave the path by gathering detailed molecular data from individual patients (The 100.000 Genomes Project, 2017), (Telenti et al., 2016). Closer to the engineering of artifacts, attempts are currently already undertaken to develop Digital Twin models of the heart (Scoles, 2016). With the availability of high throughput sequencing technologies and of wearable devices, multi-dimensional molecular pictures of normal patterns can be developed at the individual’s level. Examples in this direction are a project by a Google spin-off that will track ten thousand healthy American individuals for their genome, microbiome, physiological parameters captured by a wearable device, life style and well-being (Project Baseline, 2017).

The concept of Digital Twins therefore provides a very viable conceptual instrument for analysing the impact of individualized *in silico* models on key concepts in healthcare. It does so for multiple reasons. Firstly, the perspective taken in contemporary medicine is that of rational maintenance, optimization and even design of (very complex) bio-physical systems. Interventions in both engineering and medicine can be considered as engineering actions. Probabilistic models of human individuals in personalized medicine aim at supporting the engineering of a healthy status. This includes an approach analogous to predictive maintenance in industry. Molecular biomarkers can provide an early identification of upcoming disease states, even before the disease is manifest. Interventions can then be done to restore the system to a healthy state. Further along the same lines, human enhancement scenarios implicitly assume that humans are (eventually amongst other things) biophysical system of which the components and the functioning can potentially be understood in terms of mechanistic processes, and are therefore amenable to engineering of current features, and the engineering of novel ones. Secondly, these activities in both fields are guided by big data and by mathematical models that represent *one individual* person or artifact. In both engineering and medicine there is a strong belief that interventions will be more precise and effective, when individualized mathematical models are used that capture the actual status of one particular artifact or person over time. Models of artifacts are evidently much more comprehensive than models of an organ or of the metabolic status of a person. Artifacts have building plans and are much less complicated than human beings. Models in medicine are still very partial and coarse grained, but nevertheless already show effectiveness, as can be seen in the field of cancer treatment. By combining various types of omics-levels one can anticipate that a much higher level of predictivity can be achieved than when using only single data types, like genomic data.

## Digital Twins and the Concepts of the Normal

Digital Twin approaches in health care have the potential to vastly increase the resolution and the comprehensiveness at which one can define normality and disease. The ‘virtual self’ models will provide a detailed map that allows to better pinpoint deviations from the normal. This ‘normal’ or healthy state can be defined at a high resolution and in multiple data-dimensions, using molecular, phenotypic and behavioral level over a person’s life time. Natural variation amongst individuals, which make it otherwise difficult to pinpoint what is exactly normal, can be mapped in this high dimensional space. Heterogeneity in data acquisition is replaced by regular measurement of parameters over one’s life time. Such approach will allow to obtain a much sharper statistical definition of the normal or healthy state, and likewise of disease states or disease susceptibilities. Confounding factors like age, lifestyle, and genetic background can be take into account in such models.

High resolution models of what is normal or healthy constitutes the cornerstone of upcoming personalized medicine approaches. A detailed picture of the healthy assumedly allows for a better identification of potential or actual disease states that need to be remediated. For example, assessment of which particular chemical is optimal to treat a cancer in a specific patient requires classification of that cancer by its driver mutations. This implies a precise understanding of how a healthy genome looks like, and which deviations from this normal situation are harmful. The approaches though often base the concept of the normal on the population, not yet on the individual. Early initiatives like the Framingham Health Study used physical examinations and lifestyle interviews on a set of healthy individuals. These studies played an important role in understanding the impact of lifestyle on cardiovascular diseases (Framingham Heart Study, 2017). Population genomics studies sequence large amounts of citizens to infer genetic diseases, and by consequence build a picture of a healthy genome. Initiatives like the Metagenomics of the Human Intestinal Tract (MetaHIT), the Human Microbiome Project (HMP), and Chinese diabetes consorts reported on microbiomes of healthy individuals (Lloyd-Price et al., 2016). Multi-dimensional molecular pictures of healthy individuals are being pioneered (Project Baseline, 2017).

Next to being defined at high resolution, the normal will also be truly *personalized*. It will be based on both the disease and healthy statuses of a particular individual. To the extent that physicians already tailor treatments to the medical history and actual status of their patients, one can say that medicine has always been personalized (Brenner, 2012). This personalization though relies on coarse grained categories, plus a picture of the past *disease* states of a particular person. Digital twin approaches in health care will heavily rely on a detailed picture of the *healthy* state of an individual, not merely on a record of disease states. ‘Normal’ in this context refers to the typical molecular, physiological and behavioral patterns observed in the individual, interpreted against the backdrop of the patterns observed in the entire population. Blood pressure readouts provide a simple illustration of this point. The sphygmomanometer is available for more than 100 years, nevertheless there is not yet a clear understanding of what is a ‘normal’ blood pressure. One of the reasons is that this cuff-based blood pressure determination method results in sparse measurements over a person’s lifetime. (Steinhubl et al., 2016). This makes it impossible to assess the impact of day or night, age, caffeine consumption, stress conditions, and so on. The result is improper management of hypertension in many cases. Wearable devices nowadays can monitor an individual’s blood pressure continuously. A “virtual medical assistant” has been proposed that uses machine learning to mine these data streams and identify the blood pressure trends that are unique to that particular person. Such information can provide an individualized concept of what is a normal blood pressure, against the backdrop of trends observed in people with similar age, life style, etc. (Steinhubl et al., 2016). Similar approaches are relevant for molecular biomarkers. Identification of the risk to chronic heart failure can benefit from serial measurements of biomarkers over time, rather than from single values (Miller and Jaffe, 2016). The Digital Twin approach is in contrast with current normal function accounts that define a normal or healthy state based on statistics derived from large cohort studies. As is clear from the example of blood pressure, the ability to define what is normal based on an *individual*’s detailed history results in a very different concept of the ‘normal’ as derived from population studies. Digital twin models will be continuously fed with all types of information during the lifetime of a person. This will allow to determine what the statistically normal patterns are *for that person* for a manifold of parameters. These normal patterns for the individual might well lie out of range when compared to the ones observed in population studies. The normal will be individualized.

Thirdly, Digital Twin models will make an individual’s molecular and physiological makeup – which is currently hard to gain access to – transparently accessible. This will allow for comparing normal patterns across individuals with much greater ease and in great detail. The multidimensional space of properties across Digital Twins can be used to cluster similar individuals. Currently comparison with the normal range is mainly based on age and gender. One can expect that a high-resolution picture will lead to a great heterogeneity of types of human beings, each of them characterized by their own normal patterns. This effect already becomes apparent at the genomic level. High resolution genomic sequence data of multiple individuals revealed that human genomic variation was larger than originally anticipated (Telenti et al., 2016). Variation in genomics regions that were previously perceived as junk seemed to have functional significance when having more data at hand. Similarly, it has been suggested that there might be a manifold of healthy states in human microbiomes, and therapy entails moving the composition of the microbiome toward one of these healthy attractors (Lloyd-Price et al., 2016).

Digital Twins therefore will not only result in a better quantitative resolution when defining health and disease. The fact that Digital Twins reflect the status of individuals, and allow for a transparent comparison of these individuals, leads to a conceptual change in the distinction between health and disease. The transparency in the heterogeneity of what is normal raises the question on whether natural levels are optimal and are prone to engineering (Kahane and Savulescu, 2015). What previously was regarded as healthy, i.e., the absence of any obvious disease indications, can lose its unproblematic character in view of this transparency. Gradations in levels of ‘healthy’ will become pronounced against the backdrop of this data landscape. The healthy state can now potentially be perceived as a suboptimal condition, when compared to others in the population. A condition that requires remediation. Next to this, the healthy state can become a state of ‘symptomless illness,’ because the data allow to infer likelihoods of developing diseases. Individuals with a ApoE-4 allele for instance have a higher likelihood of developing Alzheimer’s disease (though they might never develop the disease during their lifetime). The statistical character of these inferences can transform ‘health’ into a series of disease susceptibilities, some of which can be mitigated given modifications in life style or given medical interventions. Last but not least, against the background of a Digital Twin model, the healthy state does not appear as the unproblematic natural state, but rather as an arbitrary configuration, out of many possible configurations. The engineering paradigm that comes with Digital Twins will sharply raise the question whether the healthy – normal – state indeed is optimal. It implicitly carries the question whether certain properties should be optimized or enhanced. In current health care practices, one mainly consults a physician when the normal becomes problematic and calls for action. For instance, when a disease gets manifest (e.g., experiencing a sharp pain in the stomach), or when one belongs to a certain category or has certain coarse grained indications (e.g., preventative measures to reducing the risk to osteoporosis in elderly women). In Digital Twins based health care practices, the normal may call for action.

## Digital Twins and the Concepts of Therapy, Preventative Care, and Enhancement

The distinction between therapy, preventative care, and enhancement – though intensely debated – is instrumental in decisions in health care. The distinction between therapy and enhancement was proposed as means to identify those actions that require special moral consideration, because they change the constitutive aim of our medical interventions, which is to cure. (Daniels, 2000; President’s Council on Bioethics, 2003). This viewpoint is reflected in one of the common definitions of enhancement, namely enhancement as the improvement of general abilities “beyond the species-typical level or statistically normal range of functioning” of a human being (Daniels, 2000; President’s Council on Bioethics, 2003; Allhoff et al., 2009; Menuz et al., 2013).

The concepts of therapy, preventative care and enhancement bear a striking analogy with engineering concepts, and thus offer a relevant perspective on the question whether and how Digital Twins changes concepts in health care. Engineering actions on existing systems always aim at either restoring the functioning of a system, or at modifying a system. These actions can be classified as either repair, maintenance, or improvement. In repairs the modifications address a problem, and aim at restoring a system to the normal functioning. Maintenance actions make sure that the operational life time of an artifact is optimized. Improvement actions like ‘souping up the engine of a motor’ bring an existing functionality beyond the normal, or they introduce a novel functionality. Given the strong analogies with the distinctions between therapy, preventative care and enhancement, one can expect a significant impact of Digital Twin-based engineering practices on these distinctions in health care.

Digital Twins change the existing engineering paradigm. Main elements in this paradigm shift are the high transparency of the inner status and workings of an artifact, and the centrality of each individual artifact. This changes how repair, maintenance and improvement can be done. Similarly, when a Digital Twin approach would be applied to health care, a shift in related concepts can be expected. The individualized character of the approach for instance will impact the already problematic distinction between therapy and enhancement. Such distinction namely depends on the reference taken. In the engineering cases, it is ‘the normal’ as defined in the certification or classification (e.g., of a ship or the weight of a payload, stress, torque) which helps to define the boundary between systems maintenance and problem remediation versus improvement. In a similar way, the normal in the biological realm defines the boundaries between therapy and enhancement in “species typical normal functioning” accounts (Daniels, 2000). This definition of normal functioning is often based on population statistics. When taking the individual’s normal patterns as reference in a Digital Twins approach, therapy entails the maintenance or restoration of this *individualized* normal state. It is well possible that an individual performs well in a certain trait when benchmarked against her individualized normal state, but underperforms vastly when compared to the rest of the population. In analogy with a wind turbine park, one can tune a poorly performing wind mill toward the average mills in that park, instead of bringing it back to its twin’s definition of regular performance. Or even more, one could decide to take measures to get it to the best performing mills in the park. So, even given the high-resolution picture on normal performance that can be derived from Digital Twins, the distinction between maintenance and upgrade crucially depends on the reference or baseline that is chosen, so that this distinction contains an important normative element. In the case of medical actions on humans, as has often been pointed out (Hofmann, 2017), the distinction between therapy and enhancement will not result only from a detailed observation of the state-of-affairs but also from their interplay with the realm of language and meaning. ADHD for instance has only been categorized as a diseased state in recent times (Lange et al., 2010). Nature does not come with clear categories, and is often is characterized by gradients rather than by crisp clear joints at which one conceptually can cut. As pointed out along these lines by Bostrom and others, the concept of “disease” may not refer to any natural kind and depends on the perspective taken (Bostrom, 2008). In a ‘promiscuous realism’ perspective, the human interest together with the patterns found in nature will determine where one will “carve nature at its joints” (Dupré, 1993). Along these lines, categories like therapy and enhancement do not solely reflect patterns found in the data of patients. They also reflect our normative interests and conventions.

A Digital Twin approach will lead to a high level of transparency of an individual’s molecular and physiological constitution. The impact of this molecular and physiological information is not constrained to a purely instrumental value. In the case of wind mills and jet engines, the virtual representation is purely instrumental. In the case of human begins, such transparency will make that moral distinctions can be grafted on this information. Some important moral distinctions concerning humans are rooted in, or depend on the physicalist state-of-affairs (Burms and Vergauwen, 1991). Some morally important distinctions are made based on grounds that are morally irrelevant, but are based on a material link or ‘inner structure’ (Singer, 1974). These authors illustrate the point with the example of ‘speciesism.’ Humans have the strong tendency to attribute a special moral status to human beings over animals. When having a closer look though, such fundamental moral distinction cannot be made on grounds of differences in morally relevant criteria. Animals for instance also have the capacity for suffering, and in some cases their capacity for reasoning in certain areas surpasses those of mentally retarded people or infants. The conclusion drawn from this observation is that biological origin defines who belongs to the human community, in other words the hidden inner structures and the relations of descendance that define a being as part of the natural kind “human.” Along the same lines, the growing body of knowledge on biomarkers and genes shows that data on the molecular and physiological constitution of a person can give rise to moral distinctions, when connected to properties like intelligence, entrepreneurship, susceptibility to diseases like dementia, etc. This moral load is one important reason for data privacy.

The cases exemplify that some important moral distinctions are *grafted* on structures deeply embedded in nature. If this is the case, then it is reasonable to expect that in a hypothetical scenario in which high resolution data on genetics, metabolism, life style, etc. is available for persons, and their individualized high-resolution pictures are offered by Digital Twins, we may witness changes in what we consider to be health, disease, therapy and enhancement. Consider for instance the emerging class of ‘asymptomatic ill.’ This class consists of healthy people with molecular patterns indicative of a high susceptibility to a disease, though they did not develop that disease yet (Plümecke, 2016). Now, assuming one takes some (medical) steps to prevent the disease to develop, one may wonder whether this intervention would qualify as therapy. Conceptually, it seems unwarranted to define therapy an intervention done on a healthy individual. In this respect, such preventive care interventions resemble more what from an engineering perspective would be called a maintenance intervention. However, this wouldn’t be simple maintenance, due to the specific goal for which it is done. This goal is to prevent one very specific and statistically uncommon malfunctioning or disease to occur via a targeted (medical) intervention, where the occurrence of this specific potential disease has been predicted based on a high resolution picture of the individual subject. The subject is healthy according to current health care practices, but her Digital Twin indicates a certain likelihood of developing a disease later on, therefore making that the person “is not ok.” Namely, predictions derived from an accurate digital model, being very closely intertwined with the person and her identity, will have a different load than generic observations derived from population studies. An accurate digital model of a person will not be merely instrumental in better decisions in health care interventions, but will also be part of that person’s identity. Predictions derived from such digital models will impact both the persons self-perception, and eventually societal perceptions about that person.

On the other hand, defining these interventions as forms of enhancement due to them being done on a (currently) healthy individual and/or due to them being based on information in a digital representation of the subject rather than on her actual conditions and/or being done via complex and costly interventions would not sound convincing either. After all, it is a disease that we are fighting. It may therefore well be that personalized medicine and Digital Twins will force us to further stretch or revise what we consider therapy. For instance, by accepting the idea of something being a therapy, even if done on a healthy individual based on a critical condition of her Digital Twin, insofar as the intervention is done in order to address a potential illness of the individual which is highly probable to occur. In fact, it is to strike this balance that some already use the apparently paradoxical label of “preventive medicine.” Needless to say, this is not only a conceptual but also a moral issue. Depending on whether these interventions are considered as daily care, therapy, or enhancement, different conclusions may be drawn on the question as to what extent and under which conditions they should be provided and their costs covered by a public healthcare system.

A second example of a possible shift in what we consider to be health, disease, therapy and enhancement would be life extension via anti-aging medicine. There is a high interest to develop ways to prolong the human life span, as in Google’s spinoff Calico LLC or Venter’s Human Longevity Inc. The rationale that is often used to support this type of research is a therapeutic one. Preventing diseases by making people growing old in a healthy way is better than curing diseases only when they happen to arise. Life style and genetics already result in considerable differences in life span among people, so one can expect that there are mechanisms that can be engineered in order to extend people’s life-span. Some people seem to have a constitution or habits that result in a long and healthy life. With the availability of Digital Twins, such naturally occurring people with an extremely long life-span might end up in a dedicated medically salient category. A combination of certain features in genetic makeup and lifestyle as displayed in someone’s Digital Twin namely might allow to reasonably predict their life-span. Such ability to cluster based on Digital Twins data would lead to new medically relevant distinctions between healthy persons, even without the presence of enhancement technologies. One would be able to classify a set of people as prone to lead a long and healthy live, and sets of people with normal or with short life expectancies. This medically relevant distinction between persons, again, will be grafted on top of the (statistical) patterns that are found in the population of Digital Twins.^1^ Such clustering is not possible if detailed data on the *individuals* are not available. Now, let’s imagine that, thanks to Digital Twins we come to discover with some precision which life-styles are typical of people in the class of long-livers, for instance a certain diet or a certain regime of physical activity. Let’s also assume that based on this knowledge one would gradually manage to move more people into this class. This could be done for instance via the advertisement, possibly the nudge or any other set of psychological or economic incentives to live according to these healthier life styles. Again, the question arises as to whether a life extension achieved in this way would count as therapy or enhancement. On the one hand, one may not categorize this as human enhancement. The deviation from the norm can be for the individual, and still be in the normal life expectancy range of the human species as a whole. Moreover, if a group of people starts to live whatever happens to be the life-extending life-style and thereby lives longer, this would hardly be considered enhancement. Living a healthy life is the paradigm of a health improvement that does not qualify as an enhancement (or therapy, for that matter). However, one may argue that there is a crucial difference between this scenario and the scenario that involves Digital Twins and an explicit policy of incentives. Here it can be said that a certain individual’s or group’s life extension has been achieved *by design*; because of the kind of knowledge provided by the data of the Digital Twins (high resolution, etc.), and because of the systematic, deliberate targeted policy that this knowledge has allowed for. The intertwinement of Digital Twins with a person’s identity will add to this: the transparent model allows for design operations, that then get reflected in the person via medical or life style modifications. In other words, whereas the means used to achieve life extension – food, physical activity – clearly fall into the field of natural remedies, the broader process of scientific acquisition of data and of (social) design of which they are part may turn the process into a form of engineering, and therefore, arguably, of human enhancement. In fact, if the same group of people would obtain the same life extension effect, but this time because they have the financial means to access some complex biotechnological interventions, intuition would probably lead us to classify this as enhancement. The reason is not merely that such a radical intervention surpasses a normal range derived from the distribution over the entire population. The reason to categorize this as enhancement has to be, first of all, with the *explicitly* engineering nature of this intervention. The Digital Twin type of data-driven enhancement is to a certain extend an extrapolation of the intensive follow up of professionals in sports. In the case of these athletes, measuring and tracking of all types of parameters, and the resulting continuous optimizations of life style, diet and supplements, can provide a vast competitive advantage over other athletes.

Certainly, the fact that such life extension would be achieved via *costly* technologies, would also have a symbolic boundary surpassed. It would impact the way we think about humans and aging in general. It is a vastly rooted principle in human societies that the wealthy and the poor face the same facts of life: they grow old and die. Access to health care, nutrition, housing, etc. evidently can contribute to a longer life. But biologically speaking mortality *per se* is indifferent from human action. This biological fact is rooted in culture and society since the dawn of mankind. Technological modification of this process would not only result in a biological quantum leap, but also in a quantum leap in meaning. The concept of what it is to be human may fundamentally change by means of advanced life extension technologies (Temkin, 2011). The premise that “all humans are mortal” then will not hold true for all men to an equal extend anymore. Some will be less mortal than others due to technical means, eventually because of their financial means. In this case, the transgression that determines whether a modification is an enhancement therefore is not just a quantitative change in a certain feature, but also a transgression in the domain of meaning, that is grafted on a technological modification of biology. This fact holds true whether or not it concerns radical transformations, although radical transformations probably carry a higher likelihood to affect existing symbolic distinctions more harshly.

## Digital Twins and the Ethics of Human Enhancement

So far, we have used human Digital Twins – the assumption that one is in the possession of a data magnifying glass, that gives a detailed account of the molecular, phenotypic and life-style history of persons – as a conceptual tool to understand an existing trend in medicine, and to start a reflection on the potential conceptual implication of this trend on our understanding of the categories of health, disease, and enhancement. In this last section, we use Digital Twins to explore some possible ethical and societal implications of this trend.

A popular line of argumentation in favor of the *prima facie* moral acceptability of human enhancements starts from the observation that humans already use enhancement techniques, albeit low-tech ones. Athletes for instance improve their performance via physical exercise, a special diet, and a regular life style. With the introduction of wearable health monitoring devices this type of improvements becomes supported by real time data from the individual athlete. The improvement obtained by training and dietary schemes might be the same as the improvements obtainable via pharmaceutical means, both based on these early stage Digital Twins. The aims and the factual outputs are similar, maybe even at the molecular level, which might lead to the welfarist position that therapy and enhancement are equally acceptable means to increase welfare (Giubilini and Sanyal, 2015). As outlined above though, the acceptability of the approach is not merely rooted in the data, but in the distinctions made at the level of meaning. Human enhancement achieved via technological means or programs based on Digital Twins may be seen as specifically problematic because of this. By using pharmaceutical means, an athlete may transgress a certain symbolic boundary that is institutionalized in her sport for a long time. It is exactly the transgression of this symbolic boundary that makes the athletes act problematic, not merely the result in performance. Let’s assume that a society rethinks a marathon, now entailing the usage of tailored pharmaceuticals based on the runner’s Digital Twins, as a means to boost runners performance. One might consider the resulting contest as morally acceptable if no transgression at the level of meaning would be involved. But the participants of this activity would engage in something that is different from what we now call a marathon. The constitutive rules are changed. We could also think about introducing a rule in chess (and leave other rules unchanged), that allowed a knight to jump twice in one turn. Since many human activities are defined by their point and meaning and embedding in a practice that is governed by formal or informal rules, they would engage in a very different type of activity (Whitehouse et al., 1997; Santoni de Sio et al., 2016). This is a general point that goes beyond the sports example.

Egalitarian concerns constitute one of the main bioconservative arguments to caution enhancement. The fear is that human enhancement technologies might lead to different classes of people, and therefore have a disruptive effect on our democratic institutions (Fukuyama, 2002). Along these lines, human enhancement technologies can be thought of as increasing the already existing diversity among human beings. People already differ in strength, health, intelligence or longevity. When such differences would be available as quantified properties in a person’s digital representation and available to the entire community for consulting, that evidently in itself carries the danger of discrimination and of the constitution of novel classes. This may create a crucial complication for the realization of the ideal of human enhancement as a social equalizer. Consider, for example, cognitive enhancement. Enhancers, unlike natural talent and capacities, would be at least in principle available to everybody in the same way. One therefore can argue that enhancers are potential social equalizers, counterbalancing the individual differences that are randomly assigned by the natural and social lottery (Savulescu et al., 2004). However, it turned out that individual differences matter also for the functioning of enhancers (Husain and Mehta, 2011). This doesn’t necessarily mean that cognitive enhancers may not work for a certain category of people (though it may well be the case). But it certainly means that a big quantity of individual data is needed to fine-tune the treatment or the enhancement. Digital Twins have therefore great potential to make enhancements more precise and effective, if the assumptions behind personalized medicine prove to be correct. This holds true not only for cognitive enhancement, but for all sorts of therapy and enhancement. This necessity of acquiring a massive amount of data about the individuals may introduce new issues of equality that may counterbalance the desired equalizing effect.

It hints at the fact that not the enhancements themselves, but rather the sheer availability of a vast amount of data like those of Digital Twins coupled with the human tendency to attribute meaning to patterns in data may give more concerns for equality. Digital Twins thus can sharply raise the question of distributive justice. One needs to determine whether the development of costly digital representations will be purely market driven, or whether compensation mechanisms need to be implemented for the least well off. One also needs to define which resulting possible health care interventions (be it therapeutic, preventative or enhancement actions) will be supported. Next to this, governance mechanisms will be needed for safeguarding the rights of persons that have Digital Twins. Such governance mechanisms can draw from how for instance biobanks or medical databases are designed, regulated, inspected, etc. The governance structures should for instance ensure transparency on how the Digital Twins are used, protection of the data, and a fair distribution of the benefits derived from people’s personal biological information. Data protection will be a key instrument to mitigate some of the potentially negative effects. Privacy concerns that were raised in the context of genomics will be even more relevant in the case of Digital Twins, since the combination of multiple layers of biological and behavioral data will be much more telling about a person than genomics data alone. Given also the engineering analogy that is closely related to Digital Twins, privacy will be instrumental in avoiding that persons will be on a same par as designed objects, *vis a vis* their twins. In other words, privacy will avoid blunt comparison of human Digital Twins and therefore the grafting of symbolic distinctions on top of these data.

However, this may create a trade-off or even dilemma between equality of capabilities of people to lead the lives of their choice, versus equality of privacy. In order to grant everyone access to medical treatments, distributing pills or medical devices may not be enough. In this ‘virtual patient’ scenario it is a prerequisite to collect everybody’s data and to create a Digital Twin for everybody. Personalized medicine will probably increase the cost at the individual level, when compared to off-the-shelf pills. Next to that, there will be differences in people’s capacity to protect their data, due to differences in information about the risks, and differences in their contractual position in the “negotiation” about the use of their data. This is a concern for the standard reasons about (medical) data protection (van den Hoven, 2008). But it also raises a new, specific, issue. Bioconservative fear of a class of biologically privileged persons might realize without any technological intervention; the mere existence and knowledge of one’s Digital Twin may create discrimination of the real people of which the twins are a digital representation. Self-fulfilling prophecy mechanisms similar to the ones active in the financial sector can come into play: the mere fact that other people or institutions think that you are going to be sick or weak or short-lived may make you sick, weak or short-lived. Much in the same way in which the mere fact that you are thought to be insolvent may eventually leave you broke. This marks an important difference between the use of Digital Twin in engineering and in medicine. The social and symbolic dimension in the human realm create a new layer of complication and potential ethical issues. A Digital Twin for a human may be not just a powerful tool to improve one’s physical condition. It may also be a second self who can – metaphorically speaking – rise up against its biological counterpart; or, more prosaically and realistically, being the source of serious moral damage for the real person. In this way, it may be the case that the only way to achieve equality of capabilities would be by creating data which may in turn be used to penalize some groups or to create new forms of discrimination.

The engineering approach that is inherent to Digital Twins also sheds a new light on current health care values, and opens the route to a whole new range of values. In current health care, where in most cases only a low-resolution picture of the disease trajectory of a patient is available, regular health care values that apply are autonomy, beneficence, non-malfeasance and justice (Timmermans et al., 2011). All these values will face different concretizations in case Digital Twins become available. Distributive justice for instance will be challenged due to the high resolution with which one can suddenly identify differences in constitution and capabilities among people. It will sharply raise the question on which conditions are to be treated in order to compensate for bad luck in the natural lottery. The value of autonomy will have to be implemented in view of a strong dependency of a digital model. Given a close link between the digital model and the corresponding individual, question is to which extend the patient will be able to make autonomous decisions on what is good or bad for her, and to which extend this is determined by the algorithms that claim to propose the most optimal solution based on the data at hand. ‘Dataism’ in this context might become a new form of medical paternalism. Patients thus will have to develop a proper relation toward their Personal Digital Twin, and develop the capacity to make informed decisions in view of strong data-driven personalized models.

Moreover, with the availability of detailed molecular data of novel engineering methods to impact biological systems (e.g., engineering germlines or somatic cells via CRISPR/cas), a whole range of values need to be decided upon. Examples are the efficiency of the engineering actions, the effectiveness of the design, the competitiveness of the design versus other designs. The question is then which enhancements to favor, and how to make the engineering decisions. Engineering in general requires decisions on which values to include in the design or the optimization of a system, and which values to maximize (van den Hoven et al., 2012). Value-sensitive design approaches in engineering make explicit which values are implied in the technical development of an artifact, and try to overcome moral dilemmas by design. Given the analogies with engineering, this approach can also provide relevant insights in the field of personalized medicine and Personal Digital Twins. The trade-off between equality of access to (personalized) medicine and risks of data-based discrimination is one example of a challenge that value-sensitive design may face in this domain.

Next to this, the results of medical engineering actions are intrinsically positional, as they are in the economic context of engineering artifacts. It is not the available quantity of the services that determines their value in the market, but the extent to which others have *no* access to them. If a small group of people has access to life extension products, these products will have a much higher value to them than in the case all members of a society have equal access, since in the first case it provides them a significant competitive advantage over others. The rationales for pursuing enhancements will be colored by this positional character. Individuals for instance can aim at enhancements with personal flourishing as underpinning motif (e.g., ability to even more enjoy their swimming experience), but more likely they will be driven by competitive motifs (outperform others that score less on the swimming property). A different effect is that enhancement actions may lead to an impoverishment, by focusing on certain traits and neglecting others. Since enhancement can be considered to be an engineering optimization problem, one needs to decide which optimizations to pursue. It might well be that improving an athlete’s performance will for instance lead to a decrease in longevity, or that an improved feeling of contentment leads to a decrease in entrepreneurship. Digital Twins have the potential to make these tradeoffs transparent.

Rationality has limits, and this point is often pivotal in bioconservative perspectives on human enhancement (Giubilini and Sanyal, 2015). Reason proves to be an instrument with very limited capabilities when it boils down to predicting the future. Predicting the consequences of radical enhancements is therefore merely impossible. It even proved to be difficult to assess the demographic effect of simple and un-invasive technologies like the prenatal determination of a child’s sex (Fukuyama, 2002). Hottois (1996) stressed the point that our complex bio-physical world *brings about* the future, and that these dynamics can only be captured to an extremely limited extend via reason and via our systems of language and meaning. In this perspective, one cannot fully anticipate the future impact of current human enhancements, whether they are disruptive or gradual. This lack of long term predictability not necessarily implies that enhancement actions should be banned. One can accompany the process of making bio-physical modifications, with deliberation about meaning, value, risks, etc. Since Digital Twins constitute a bridge between the bio-physical world and the world of language and meaning, they can become an important technical platform for enabling such techno-moral accompaniment. The data in Personal Digital Twins reflect the operational character of reality. These data are read-outs of the metabolic composition of the blood at a given point in time, the genomic code, the history of blood pressure and of physical movements of the body, and so on. As such, these data are an intermediate stage between the operational realm of the biophysical reality, and the realm of symbols, language, and meaning. Availability of these data provides us with a substrate to graft symbolical distinctions and meaning on structures that are present in the bio-physical world. Digital Twins, be it as conceptual tool or as emerging technology, can therefore be a tool for moral accompaniment of technological evolutions. They can be one element, among many others, in an effort to realize a Responsible Innovation in this domain, and aid both in understanding and in shaping the continuous interactions between engineering actions in the bio-physical world, and the world of values and meaning.

## Conclusion

The Digital Twins concept provides a solid thought instrument to analyze conceptual and ethical aspects of future healthcare and human enhancement. It does so by putting enhancement against the backdrop of individualized high-resolution data of people’s molecular constitution, physiology, life style, and dietary habits. Next to that, Digital Twins are an emerging field in medicine, that has the potential to become the playfield where therapy and enhancement are explored. Comparison between Digital Twins in entire populations allows to get a much sharper idea on health versus disease, and by consequence sharpen the debate on therapy versus enhancement. Digital Twins have the potential to be a rich source for identifying novel and effective engineering routes, both for therapy and enhancement. As such, Digital Twins can allow to identify physical well-being parameters that one would prefer. Digital Twins also have the potential to impact a person’s identity, since meaning can be assigned to the patterns in the data. The engineering paradigm inherent to a Digital Twins based health care will raise novel ethical, legal and social issues for therapy and enhancement. Digital Twins for instance can challenge equality, even without the application of enhancement technologies. The differences between persons can be sharply defined and made extremely transparent based on the differences in their compiled information, leading potentially to segmentation and discrimination. Personal Digital Twins are an asymptotically data-intense scenario that clarifies the importance of governance concerning the production and use of personal biological and life style data.

## Author Contributions

KB and JvdH conceived of the presented approach. KB took the lead in writing the manuscript. FSdS provided contributions to the sections on the ethics of enhancement and the distinction between therapy and enhancement. All authors discussed the approach and contributed to the final manuscript. JvdH supervised the work.

## Conflict of Interest Statement

The authors declare that the research was conducted in the absence of any commercial or financial relationships that could be construed as a potential conflict of interest.

## Footnotes

1 Some ethical implications of such scenarios are discussed in the last section.
